# Two Layer Sheets for Processing Post-Consumer Materials

**DOI:** 10.3390/polym14081507

**Published:** 2022-04-07

**Authors:** Lisa-Maria Wittmann, Dietmar Drummer

**Affiliations:** Department of Mechanical Engineering, Institute of Polymer Technology, Friedrich-Alexander-Universität Erlangen-Nürnberg, Am Weichselgarten 10, 91058 Erlangen, Germany; dietmar.drummer@fau.de

**Keywords:** multilayer sheet, extrusion, post-consumer material

## Abstract

An increasing percentage of post-consumer materials (PCR) is becoming more and more important in all processing methods in polymer technology, also due to the lack of raw materials and political demands. Very special requirements are placed on material properties such as viscosities in extrusion. Low viscosities and the presence of particles affect extrusion in a negative manner. In this study, the use of multilayer sheets is determined to both ensure extrudability and contribute to a significant improvement in surface qualities. The focus is placed on the influence of viscosity and particles on mono- und multilayer sheet quality. Therefore, two different virgin materials with a melt flow rate (MFR) of 3 g/10 min and 6 g/10 min and two different PCR materials with a MFR of 16 g/10 min and 50 g/10 min are processed both in monolayers and in two layer sheets. Rheological investigations, optical analysis, and film thickness distributions are used to show the relationship between matrix viscosity and particles. The results show that the use of multilayer extrusion can improve both extrudability and sheet quality, so that multilayer sheets can offer a significant potential in the processing of PCR materials.

## 1. Introduction

Coextrusion produces multilayer sheets in one single continuous process [1]. Two or more polymers are combined to a multilayer sheet. Different die systems can be used for coextrusion processing, namely the (multi)manifold die and the feed block system. While the distribution in the (multi)manifold die is similar to that of a monolayer die, and the individual materials only have a short common dwell time in the die, in the feed block system the materials are already stacked in the feed block. In the feed block, the materials thus have a relatively long common flow length, which can cause flow instabilities in the case of high viscosity differences. Therefore, viscosity is one determining factor for layer distribution. Wave-like instabilities and encapsulations are the main occurring instabilities that originate in the die [2]. At the interfaces between different viscous materials, flow instabilities occur. The high viscous material is encapsulated by the less viscous material. (Multi)manifold dies are suitable for the extrusion of materials with quite different viscosities, since the shorter dwell time means that less flow instabilities can develop in the die. The disadvantages of the (multi)manifold system are the high investment cost and the higher adaptation effort to existing technology compared to the feedblock system [3,4]. Thus, feedblock systems are mainly used for coextrusion.

Evaluating the flow processes in multilayer systems is in general a challenge [5]. Han and Shetty [6,7] carried out some initial investigations on the influence of viscosity on the flow process in coextrusion. The velocity distribution, the shear rate profile, and the shear stresses can be predicted by rheological properties of the individual materials [6]. Martyn et al. [8] found out that the ratio between major and minor flow of the extrudable low-density polyethylene (LD-PE) material should be 2:1 in order to prevent waviness instabilities. The thickness ratio and the viscosity difference are responsible for the thickness distribution in the multilayer sheet. Instable ranges are developed for low thickness ratios as well as for high viscosity differences [7]. For highly viscous materials, Dooley [9] postulated that the ratio of the viscosity difference should not exceed four in order to prevent encapsulation. Furthermore, for polymers with broader molar mass distribution, instabilities develop more quickly [10].

Despite the above-mentioned challenges, the application of multilayer sheets covers a wide field of products. The use of multilayer sheets is especially well established in the food packaging sector. In this case, barrier properties can be adjusted by combining different materials and thus generate highly complex functional films [11], whereas polypropylene (PP) or polyethylene (PE), for example, are impermeable to water vapor but are permeable to oxygen or carbon dioxide. For creating gas-tightness, an additional polyamide layer is included in the multilayer sheet, which has to be attached to the polyolefin-based materials by using an adhesion layer. Coextruded sheets are also applied in the field of technical parts, for example, to improve optical properties or to increase UV resistance [3]. Multilayer sheets are also used in recycling processes. Multiple processing is accompanied by a change in rheological and structural properties, some of them unfavorable for extrusion processing.

However, the rheological properties of aged polymers can present special challenges for coextrusion. There are many investigations discussing the influence of multiple processing on the material behavior of PP. Thus, for example, a decrease of viscosity can be found during multiple processing. The reason can be the decrease and narrowing of the molecular weight [12]. Low viscosity is caused by chain scission, which is the dominant PP degradation mechanism [13]. The dimension of the chain scission is dependent on the molecular weight [14]. In particular, rheological parameters such as the cross-over point of the storage and loss modulus can provide information about, for example, the chain length of the polymer [15]. Comparing two polymer types with the same molecular mass distribution but different molar mass, it can be seen that the cross-over point between storage modulus G’ and loss modulus G’’ is lower for polymers with a higher molar mass. This is due to the fact that the molecules are less flexible and thus are therefore less mobile. The lower the molar mass, i.e., the shorter the chains are, the more the cross-over point shifts to the right, because the short chains remain mobile even at higher frequencies. [15] The shorter molecular chains and the smaller molecular weight contribute to a less pronounced shear rate-dependent viscosity behavior [16,17].

As there are only investigations dealing with new materials that are aged by multiple processing, investigations discussing material behavior of postconsumer (PCR) materials are mainly unknown. Material degradation as well as the influence of particles remaining even after reprocessing have not been considered yet. In this investigation, 2-layer (layer A and layer B) containing PCR material was applied. The sheet homogeneity as well as the particle influence on the surface quality were examined dependent on the material viscosity. Therefore, new PP types with different MFR values were used as stabilizing and covering layer (layer A) in this investigation. The MFR values of layer A were 3 g/10 min to 6 g/10 min. The PCR material (layer B) used had a MFR value of 16 g/10 min and 50 g/10 min.

## 2. Materials and Methods

### 2.1. Materials

Different types of commercially available PP were used to evaluate the influence of polymer viscosity on the sheet homogeneity, the surface quality, and the particle distribution. The coextruded sheets exist of layer A and layer B. The materials of layer A were selected so that they would also be suitable for a following thermoforming process. The PP-homopolymers (HP525j (3n) and HP501l (6n)) are suitable for extrusion and thermoforming according to datasheets of LyondellBasell (Industries N.V., Rotterdam, The Netherlands). The material of layer B is a PCR material (QCP P (16r), QCP T(50r)), which is again supplied by LyondellBasell. According to the data sheet information, the PCR material receives at least 95% of recycled material from pre-sorted municipal plastic waste and has a filtration level of 150 μm. Table 1 lists the materials and the abbreviations used in the following. The abbreviations are defined by the MFR value and whether the material type is new (n) or PCR (r).

### 2.2. Sheet Extrusion

Two identical 25 mm twin screw extruders (ZK 25 P (COLLIN LAB & PILOT SOLUTIONS GmbH, Maitenbeth, Germany)) were used to extrude the mono- and 2-layer sheets with different layer thickness ratios. The coat-hanger die has a width of 250 mm and an adaptable thickness adjustment. An increasing barrel temperature profile towards the die was used, so that the die temperature was set to 180 °C. The total rotational speed of the melt pumps of extruder A and B was 54 rpm. The corresponding melt pump settings for the different layer configuration can be found in Wittmann and Drummer [18]. Table 2 summarizes the thickness ratios, the melt pump settings, and the extrudable sheet configurations.

It was possible to produce all layer ratios (A100_B0; A70_B30; A50_B50; A0_B100) for the selected material combinations.

The extruded sheets had a total thickness of 550 μm. As two layer sheets were extruded, the following abbreviations were used:

- A70_B30: Layer A has a thickness percentage of 70% of the whole 2-layer sheet and layer B only 30%.
- A70:3n_B30:16r: In this case material, 3n (HP525j) is used as the material of layer A with a thickness percentage of 70%. Layer B consists of 16r (QCP P) with a thickness percentage of 30%.

### 2.3. Material Characterization

#### 2.3.1. Thermal Analysis—Differential Scanning Calorimetry DSC

Differential scanning calorimetry (DSC) measurements were performed using a Discovery-2500 TA instrument (Waters Corporation, Milford, MA, USA) according to DIN EN ISO 11357-1 in order to analyze the melting and recrystallization behavior of the different PP materials as well as to detect material impurities. In the present study, the sample weight taken was approximately 2–5 mg. The sample was heated together with a reference specimen from 20 °C to 200 °C at a heating rate of 10 K/min under a nitrogen atmosphere. This was followed by an isothermal holding time of 30 s. The subsequent cooling to 20 °C was carried out at a cooling rate of 10 K/min. The heat flow between the sample and the reference was measured as a function of temperature.

#### 2.3.2. Viscosity Number

The viscosity number of polymers, which provides information on the molar properties of the polymer, is determined using the so-called Ubbelohde capillary viscometer [19,20]. The transit time of a defined amount of solution through the capillary is measured and compared with the transit time of the pure solvent [21]. The viscosity number is determined for PP according to DIN EN ISO 1628-3 [22]. For the determination of the viscosity number, the solvent Decalin-Irganox (Carl Roth GmbH + Co. KG, Karlsruhe, Germany) was used in this work. The dissolution process took place at 150 °C in the heating oven for approximately 300 s.

#### 2.3.3. Rheological Characterization

For rheological characterization, a Discovery HR-2 plate-plate rheometer (TA-Instruments, Inc., Waters Corporation, Hüllhorst, Germany) was used. A pellet is clamped between two axially symmetrical rotation plates with a defined preload. One plate is fixed, while the opposite plate can rotate. The rotating plate is subjected to a rotational or oscillatory movement, which induces a corresponding drag flow in the polymer. The stress response provides information about the viscoelastic properties of the polymer melt. The rheological measurements are used to determine different material characteristics. In the following investigation, the measurements were carried out in the molten state at 180 °C. The temperature selection was based on the die temperature used during extrusion. An angular frequency range of 0.1 rad/s to 500 rad/s was chosen. The cross-over point of storage and loss modulus was used to evaluate the viscous and elastic material behavior, as this point also provides information about the molar mass and the molecular weight distribution. The cross-over point at low angular frequencies indicates a polymer with long polymer chains. If the cross-over point is located on the right, the polymer chains tend to be shorter or less branched.

High-pressure capillary rheometer measurements were performed to determine rheological parameters related to the extrusion processing. A counterpressure Rheograph 75 (Goettfert, Werkstoff Prüfmaschinen GmbH, Buchen, Germany) with a 10 mm piston diameter was used to analyze the shear rate dependent viscosity. A die temperature of 190 °C was selected in order to be able to compare all materials. This temperature corresponds more or less to the die temperature during extrusion. After preheating the measuring chamber for 600 s to 190 °C, the polymer pellets were filled in several steps. Manual compression and degassing ensured a completely filled chamber without air inclusions. The ram speed was adjusted to obtain the desired shear rate. The so-called Bagley correction and the Weissenberg–Rabinowitsch correction were carried out to take into account the structure-viscous behavior of the polymer melts. The Bagley correction determines the true wall shear stress, and the Weissenberg–Rabinowitsch correction the true wall shear rate.

The shear rate values $\dot{\gamma }$ were calculated according to

(1) $$\dot{\gamma }=\frac{6\cdot \;\dot{V}}{B\cdot H^{2}}$$

where

$\dot{\gamma }—\mathrm{calculated}\;\mathrm{shear}\;\mathrm{rate}$
$\dot{V}—\mathrm{Melt}\;\mathrm{pump}\;\mathrm{volumetric}\;\mathrm{flow}$
$B—\mathrm{Die}\;\mathrm{width}\;\left(250\;\mathrm{mm}\right)$
$H—\mathrm{Die}\;\mathrm{gap}\;\mathrm{Height}\;\left(1\;\mathrm{mm}\right)$

Thus, for example, the viscosity ratio of A30_B70 is calculated by the following equation:

(2) $$\mathrm{Viscosity}\;\mathrm{ratio}=\frac{\mathrm{Viscosity}\;\mathrm{of}\;\mathrm{Layer}\;A\;\mathrm{at}\;a\;\mathrm{shear}\;\mathrm{rate}\;\dot{\gamma }\;\mathrm{of}\;60{\;s}^{-1}}{\mathrm{Viscosity}\;\mathrm{of}\;\mathrm{Layer}\;B\;\mathrm{at}\;a\;\mathrm{shear}\;\mathrm{rate}\;\dot{\gamma }\;\mathrm{of}\;150{\;s}^{-1}}\;$$

### 2.4. Sheet Characterization

#### 2.4.1. Optical Analysis

A scanning electron microscope (SEM) of type Gemini Ultra-Plus (Carl Zeiss AG, Oberkochen, Germany) was used to study the type of particles included in the PCR material. The particles were imaged at 100× magnification. Next, an energy dispersive X-ray spectroscopy (EDX) analysis was performed. For this purpose, the samples were sputtered with platinum/palladium, and the elements present were analyzed.

To count the particles existing in the sheet over a large area, sheet cuttings of 150 mm × 200 mm were photographed with a Canon EOS 5DS R (Canon Kabushiki-gais; Krefeld, Germany). The pictures of the sheets were taken on an illuminated glass plate to ensure good contrast. Afterwards the photos were binarized with the help of the software GIMP Portable 2.10.28, GNU Image Manipulation Program. Then, particle analysis of an area of 100 mm × 100 mm was performed in the software ImageJ.

In order to analyze the influence of the particles on the surface quality, sheets were also investigated with the 3D laser scanning microscope VK-9700 (Keyence Corporation, Ōsaka, Japan) with a magnification of 2.5× and 20×. A VK-X Series Multifile Analyzer 2.1.3.89 was used to analyze the particle diameter and the surface defects generated by the particles.

In addition, particles were examined under a scanning electron microscope, and an elemental analysis was performed.

#### 2.4.2. Measurement of Sheet Thickness

The sheet thickness distribution was evaluated with a Dualscope FMP100 thickness gauge measurement system (HELMUT FISCHER GMBH, Institut für Elektronik und Messtechnik, Sindelfingen, Germany). Therefore, a coordinate system was plotted in (MD) and transverse (TD) to the extrusion direction in the center of the film. The measuring points had a distance of 10 mm. A total of 10 sheets was measured for each setting. The homogeneity of the sheets was evaluated using Equation (3) as follows:

(3) $$\;\mathrm{Homogeneity}=\frac{\sum_{i=0}^{n}\;\frac{\mathrm{Standard}\;\mathrm{deviation}\cdot 100}{\mathrm{Nominal}\;\mathrm{thickness}\;\mathrm{value}}}{\mathrm{Number}\;\mathrm{of}\;\mathrm{measurements}\;}$$

According to DIN EN ISO 4591:1992-12, the thickness tolerance for sheets with a nominal thickness larger than 13 μm is ±5%.

## 3. Results

### 3.1. Thermal Analysis

**Figure 1:**
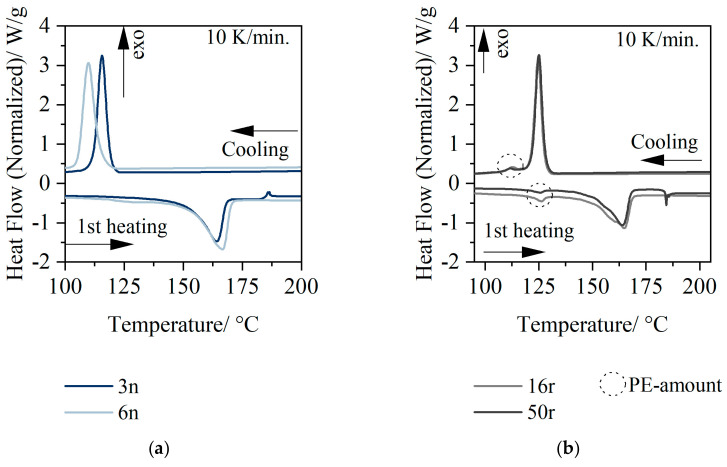
Thermal analysis of the used material: new PP (3n (HP525j), 6n (HP501l) (**a**); and PCR PP (16r (QCP P, 50r (QCP T)) (**b**).

Figure 1 shows the melting and cooling behavior of the granulates used at a heating and cooling rate of 10 K/min, since good results were achieved here according to Ehrenstein et al. [23].

The thermal analysis results showed that the melting peak temperature at a heating rate of 10 K/min for 3n was 161 °C. The recrystallization started at 121 °C. The melting behavior of 6n was similar to 3n. Recrystallization started at 117 °C.

The PCR material 16r and 50r, respectively, exhibited two melting peaks. The peak at 125 °C indicated the presence of PE parts, and the second PP characteristic peak was at 162 °C. The onset of recrystallization in the PCR material was shifted to higher temperatures (127 °C) in comparison to new PP, which can be explained by the nucleating effect of the impurities in the PP. This nucleating effect is morphologically manifested by a more finely spherulitic microstructure [24]. An estimation of the mixture based on the thermal analysis showed that the PCR material contained about 14% PE. The second small recrystallization peak indicated a low PE content. For this quantitative comparison, the melt enthalpies and peak heights were compared [2].

### 3.2. EDX-Analysis

**Figure 2:**
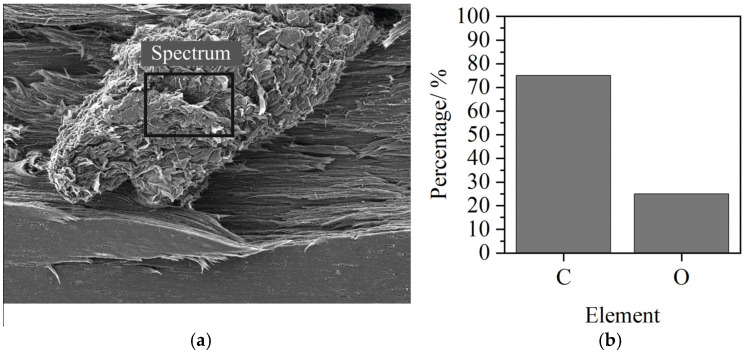
SEM of an exemplary particle (**a**) and result of the EDX analysis (**b**).

The results of the EDX analysis are shown in Figure 2.

Different particles were examined in the EDX analysis, but only the elements carbon (C) and oxygen (O) could be detected.

### 3.3. Viscosity Number

**Figure 3:**
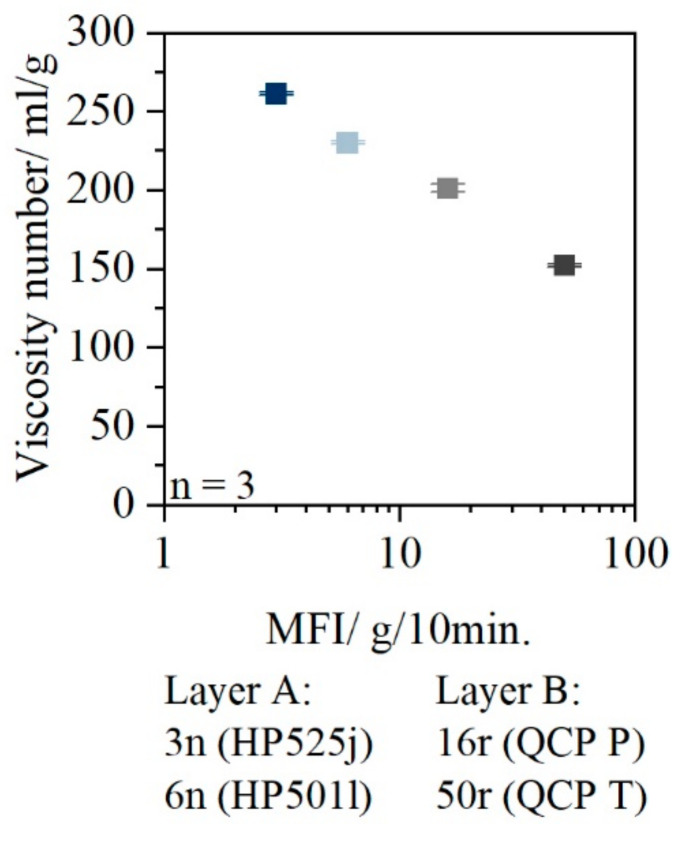
Viscosity number of 3n (HP525j), 6n (HP501l), 16r (QCP P), and 50r (QCP T).

Figure 3 shows the viscosity number of the materials used.

The viscosity number of 3n was 260 mL/g; 6n had a viscosity number of 230 mL/g. The viscosity numbers of the PCR materials used were 201 mL/g (16r) and 152 mL/g (50r).

### 3.4. Rheological Results

**Figure 4:**
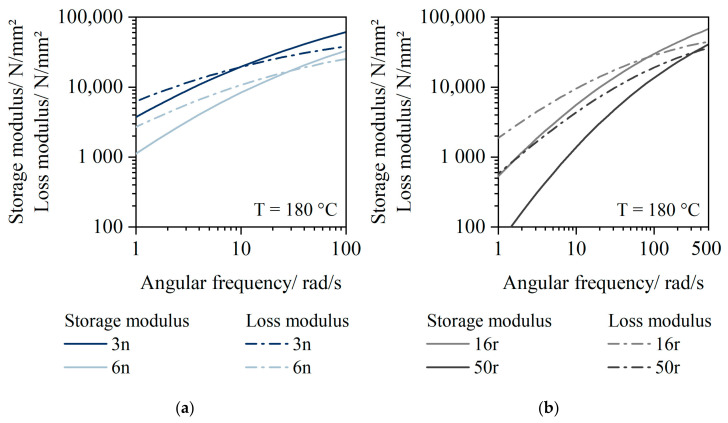
Storage and loss modulus of 3n (HP525j) and 6n (HP501l) (**a**), and 16r (QCP P) and 50r (QCP T) (**b**).

Figure 4 shows the storage and loss modulus at a temperature of 180 °C. As the granulate behaved similarly, only one exemplary measurement is shown for reasons of overview.

The plot of the storage and loss modulus of the virgin material (Figure 4a) shows that the elastic and viscous content of the material were very similar over a wide range of frequencies. The cross-over point of 3n was at a frequency of 10 rad/s, and at a frequency of 25 rad/s for 6n. Storage and loss modules of 16r and 50r (Figure 4b) show marked differences, however. The cross-over point was at 80 rad/s for 16r and 350 rad/s for 50r. The level of the cross-over point was found at a similar level.

**Figure 5:**
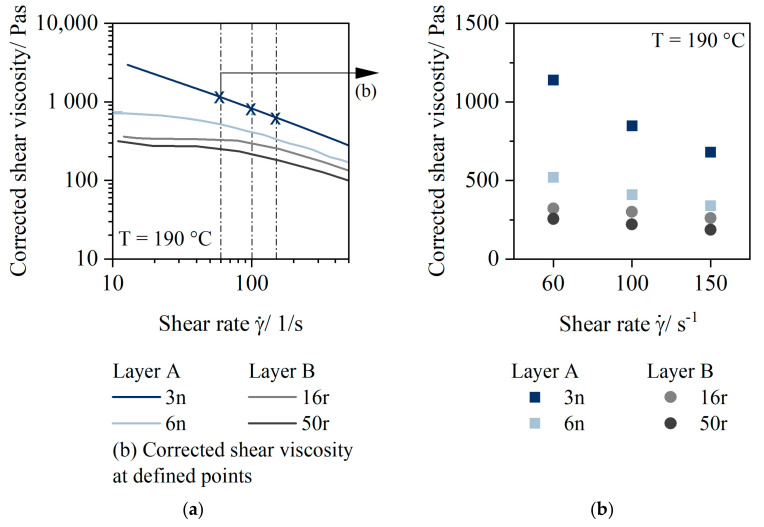
Corrected shear viscosity dependent on the shear rate for 3n (HP525j), 6n (HP501l), 16r (QCP P), and 50r (QCP T) at 190 °C (**a**). Corrected shear viscosity at the defined points of 60 s^−1^, 100 s^−1^, and 150 s^−1^ at 190 °C (**b**).

Figure 5a plots the corrected shear viscosity values of the materials dependent on the shear rate at a temperature of 190 °C. Figure 5b shows corrected shear viscosity values at defined shear rates derived from the Figure 5a.

Material 3n had a high viscosity over a wide shear rate range (100 s^−1^: 850 Pas). Material 6n had a much lower viscosity (100 s^−1^: 450 Pas). For both materials, a decrease in viscosity as a function of shear rate was evident. Shear rate dependent viscosity behavior also occurred for PCR materials, but it was much less pronounced compared to virgin materials. Thus, for example, the viscosity values for 16r were 300 Pas over the entire shear rate range of 60 s^−1^, 100 s^−1^, and 150 s^−1^, and those of 50r were 250 Pas.

**Figure 6:**
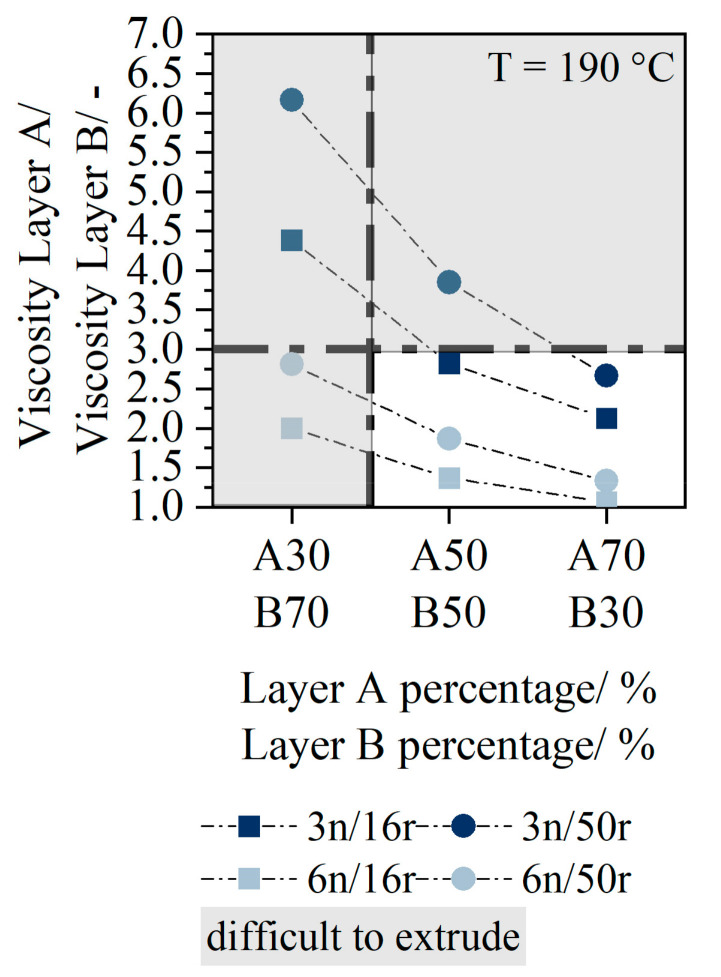
Viscosity ratio of layer A and layer B.

The viscosity ratio of the two layers A and B calculated according to Equation (2) in Figure 6 shows that the viscosity difference was significantly higher (A50:3n_B50:16r ratio = 3) when 3n was used as the stabilizing layer component compared to 6n (A50:6n_B50:16r ratio = 2).

In addition, the reduction of the percentage of layer A was always associated with an increase in the viscosity ratio. The layer ratio A30_B70 could not be extruded in any of the material combinations, since wave-like instabilities always developed in the die, as the ratio between the major and minor stream was greater than 2:1 [8]. With the layer ratio A50_B50, there was always a displacement of the low-viscosity material towards the edge of the die. However, this displacement had no effect on the film thickness distribution, as shown in Wittmann and Drummer [18].

### 3.5. Optical Analysis

**Figure 7:**
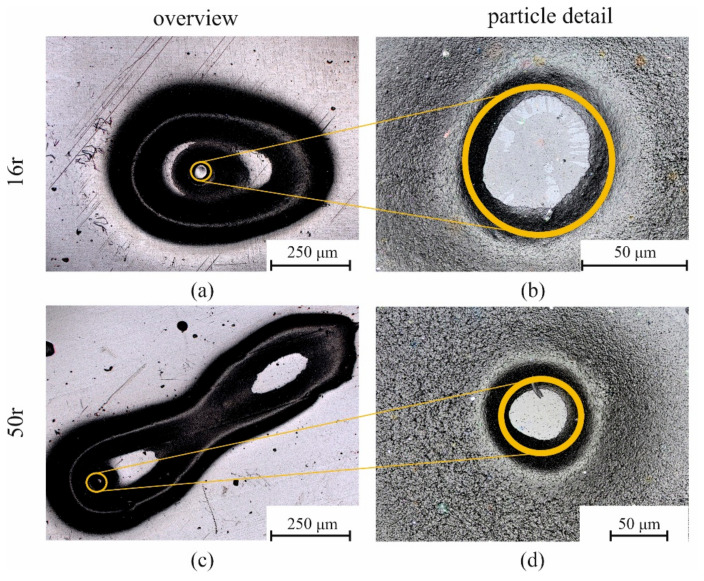
Exemplary laser scanning microscopy photos of the optical analysis for a particle in 16r (QCP P) (**a**,**b**), and a particle in 50r (QCP T) (**c**,**d**).

Figure 7 shows exemplary surface defects of the monolayers that occurred frequently for the PCR material types (16r and 50r). Figure 7a shows the extent of a particle with a size of 50 μm on the defect area in monolayer 16r. The effect of a particle in monolayer 50r is shown in Figure 7c. Figure 7b,d, respectively, show the particles causing the defects at a higher resolution.

**Figure 8:**
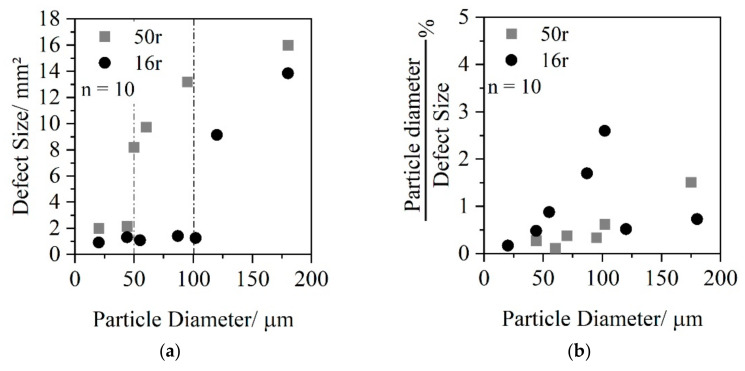
Relationship between particle size and defect area (**a**); percentage of particle size to defect area (**b**).

Figure 7a shows that the particle in the monolayer 16r caused an elliptical shaping of the defect. In contrast, monolayer 50r had two elongated elliptical defects. Since the optical illustration only served to indicate the extent to which the particles present in the PCR material affected the sheet surface, a quantitative comparison was plotted, as seen in Figure 8.

In Figure 8a, the defect area is plotted as a function of particle diameter. Figure 8b shows the particle diameter in proportion to the defect area.

For monolayer 16r, it can be seen that the defect area had an almost constant value of 1 mm^2^ up to a particle size of 100 μm. If the particle size was bigger than 100 μm, the defect area increased abruptly. In contrast, the defect area of the monolayer 50r was only constant up to a particle diameter of 50 μm with a defect size of 2 mm^2^. After that, the defect area increased continuously as a function of the particle size. Considering the influence of particle diameter on the defect area, it can be seen in Figure 8b that for monolayer 16r, there was a correlation between particle size and defect area. Up to a particle size of 100 μm, the particle size took up a high proportion of the area affected. Above 100 μm, the particle was small in relation to the surface defect. For monolayer 50r, small and large particles (0 μm–100 μm) caused similarly sized surface defects. For particles with a size bigger than 100 μm, the particle diameter was small in comparison to the defect size.

Since in the multilayer sheets the layer A enclosed the particles and no particles were on the sheet surface, the number of particles between layer A und layer B was counted by evaluating the binarized photos. As layer A was always new PP, no particles were contained.

**Figure 9:**
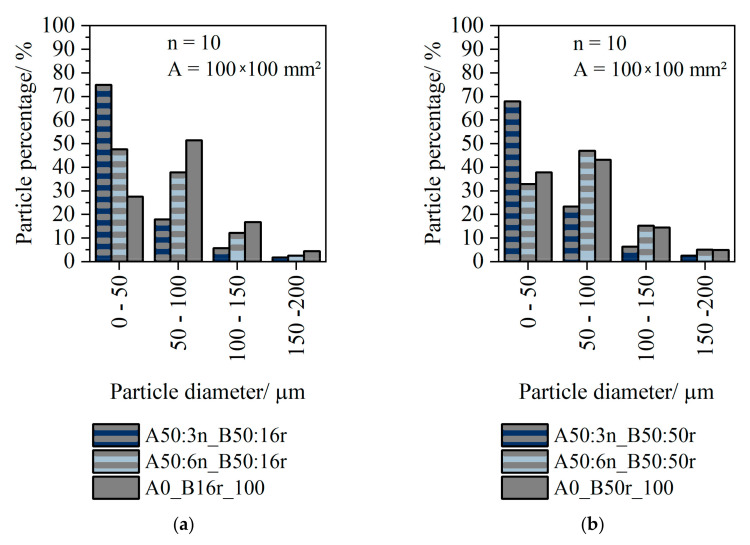
Percentage of particles in monolayer 16r and 2-layer sheets with 16r (QCP P) as layer B (**a**); percentage of particles in monolayer 50r (QCP T) and 2-layer sheets with 50r as layer B (**b**).

Figure 9 shows the number of particles present in the sheets. Figure 9a shows the particle count in the monolayer 16r compared to the 2-layer sheet. Figure 9b shows the particle number in the monolayer 50r compared to the particle number in the multilayer sheet.

Since it can be seen for both the monolayer 16r and 50r, as well as the multilayer sheets, that the proportion of particles >200 μm was negligible, this size was not be considered in the subsequent evaluation. For the A3n/B16r and A3n/B50r combination, the proportion of particles 100 μm–150 μm was less than 10%. For the combination A6n/B16r and A6n/B50r, the proportion of particles 100 μm–150 μm was around 15%. Figure 9a shows that in monolayer 16r, particles between 50 μm–100 μm represented the largest fraction of particles with 55%. Small particles (0 μm–50 μm) were present with a percentage of 25%. For 6n/16r, the proportion of 0 μm–50 μm and 50 μm–100 μm was about 40%. For A3n/B16r, the proportion of particles was 75% (A50_B50). Similar trends were also seen for the combination with 50r. The proportion of small particles increased.

### 3.6. Results of the Sheet Homogeneity

**Figure 10:**
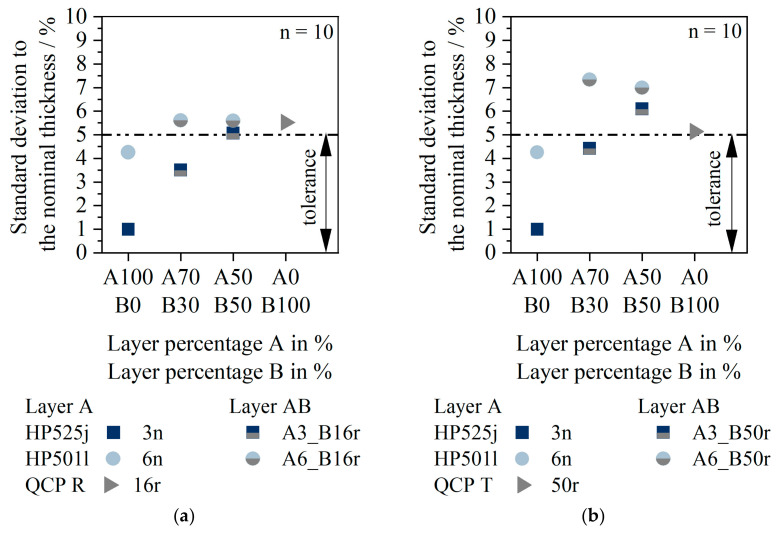
Standard deviation to the nominal sheet thickness for 16r (QCP P) as layer B (**a**), and 50r (QCP T) as layer B (**b**).

Figure 10 shows the deviation of the sheet thickness from the desired nominal sheet thickness. In Figure 10a, material 16r is used as layer B. In Figure 10b, material 50r is layer B.

The standard deviations of the two monolayers 3n and 6n were both within tolerance. Based on the standard deviation, however, it can be seen that material 3n had a significantly more homogeneous thickness distribution in the monolayer (deviation 1%) than material 6n (deviation 4%). The monolayers 16r and 50r were already outside the tolerance, although no difference between the materials was apparent.

In case of the multilayer sheets, there was a difference in the layer thickness homogeneity depending on both the layer material A and the layer material B used. If layer B consisted of material 16r, the standard deviations from the nominal thickness for 3n as layer A were about 3% for A70_B30 and 5% for A50_B50. For material 6n as layer A and 16r as layer B, the standard deviations were slightly higher. However, no difference could be seen between the two layer configurations (A70_B30 and A50_B50, respectively) for 6n as layer A.

If 50r was processed in layer B, significant differences occurred both as a function of the layer percentage and as a function of the layer material A. If layer A consisted of 3n, the standard deviation from the nominal thickness for A70_B30 was within the tolerance. If the layer proportion of B was increased to 50%, the standard deviation exceeded the tolerance (deviation: 6%). With 6n as the layer material, the standard deviations from the nominal thickness were 8% for both a low and high proportion of 50r in layer B.

## 4. Discussion

The analysis of the melting behavior shows that small amounts of PE are contained in the PP material. However, this small proportion should not be the focus of further investigations, since it is known from the literature, for example, that small percentages of PE have no influence on the forming behavior [25].

The results of the viscosity number measurement for estimating molecular degradation show that higher molecular weights are present for the selected virgin material than for PCR materials. The PCR materials show, due to the reuse, a molecular degradation as well as a lower viscosity.

The position of the cross-over point also confirms the degradation in the PCR material, as the cross-over points are shifted significantly further to the right than those of the PP virgin material; 3n and 6n thus have significantly longer molecular chains compared to 16r and 50r.

The viscosity of the PCR materials used seems to be independent of the indicated MFR value, especially at shear rates that are close to the extrusion process. This can possibly be explained by the presence of numerous short chains that slide off each other and do not need to be disentangled. The low molecular weight fractions encourage flow during the processing [15]. Viscosity at high shear rates is low (200 and 300 Pas, respectively) for both 16r and 50r. These viscosity values are very low for ensuring stable extrusion.

First, the influence of viscosity on the extrudability of the two-layer sheets and the possible layer configurations will be discussed. The resulting flow instabilities during the extrusion process due to the viscosity values are pronounced for the PCR materials in a similar way as for the virgin material [18]. The flow of the low viscous material towards the die edge results in an energetically more favorable equilibrium in the flow. The low viscosity material acts as a lubricant film for the high-viscosity layer. [15] The flow instabilities can also be observed in materials with small viscosity differences (6n, 16r). This can possibly be explained by the presence of low viscosities in the individual layers. The difference in the viscosity ratio between the two materials has to be significantly less than that required in the literature [9]. The viscosities should not differ by a factor of 3 for the same layer ratio [18]. Layer configurations with the same layer content can be produced with both a layer material A3n and a layer material A6n. However, a 2:1 ratio of melt streams appears to be valid for processing low viscosity materials as well [8].

The results of the EDX analysis allow eliminating the presence of inorganic components or metallic fillers.

The optical analysis of both the monolayer and multilayer sheets showed that the use of a multilayer sheet system can almost completely compensate any particle defects that occur on the sheet surface. The number of particles per area considered is the same for all sheet configurations as a result of the layer material B (16r or 50r) used. The extent of the defect area in the monolayers is significantly larger for 16r than for 50r. The increased defect area could be attributed to the lower matrix viscosity of 50r (1.5 times lower than 16r). In the low viscous matrix material, the particles follow the rotation of the chill-roll over a long distance, resulting in larger surface defects. Since no particle defects can be seen on the surface when using a 2-layer sheet, multilayer sheets can thus contribute to the further processing of PCR materials.

By plotting the particle fraction in relation to the particle size, it can be seen that the fraction of small particles in the multilayer sheet increases. This can possibly be explained by the evaluation method. In the 2-layer sheet, the particles are completely enclosed, and particle dimensions are partially covered by the matrix material, which could make the particles appear smaller.

The analysis of the film homogeneity shows that it is influenced by the materials used for the single layer of the multilayer sheet. The tolerance values of the PCR monolayer must be considered with caution, since the presence of particle defects can cause irregularities in the thickness measurement. The measurements were carried out at locations without particles, and deviations of up to 80 μm can occur at particle locations. The fact that the sheet thicknesses are within the required tolerances in the presence of a stabilizing layer can be explained by the covering of the particles with matrix material. The decrease in sheet homogeneity of the monolayer 3n during multilayer extrusion with a PCR material can be explained by flow instabilities occurring in the die and the flow of the low viscosity material toward the die wall caused by the viscosity ratio.

Further investigations should focus, for example, on the mechanical properties dependent on the material viscosity and the present particle as particles as well as how low viscosity influences the mechanical properties in a negative way. Since the low viscosity of PCR materials can lead to problems not only in extrusion, the following investigations will also examine the benefit of a stabilizing layer in thermoforming.

## 5. Conclusions

Different new PP types and PCR PP were used for extrusion of mono- and 2-layer sheets. Rheological measurements and optical evaluations were conducted and discussed. The major findings are summarized in the following:

- PCR PP can be extruded in a monolayer, requiring a very low extrusion temperature due to its low viscosity and low melt stability. The surface quality of the resulting PCR PP sheets is quite poor, as particles contained in the PCR material cause surface defects.
- The defect area caused by the particles increases with growing particle size as well as with decreasing matrix viscosity. At low matrix viscosity, small particles already cause larger defect areas.
- The use of a 2-layer sheet can hide the surface defects caused by particles. The layer configurations that can be produced are limited to a layer content of 70% and 50% of Layer A. Further increasing the percentage of Layer B results in flow instabilities caused by viscosity mismatch and poor melt stiffness.
- The homogeneity of the sheet thickness depends on the matrix viscosity used. The use of a higher viscous matrix as a stabilizing layer contributes to a more homogeneous layer thickness distribution, especially in the case of extremely low viscous material.
- Viscosity is a significant parameter for extrusion of PCR materials in multilayer sheets.
- Thus, multilayer extrusion can be used as a suitable method for increased processing of less viscous materials containing particles as known from recycling.

## Figures and Tables

**Table 1:** Used materials and abbreviations.

Material		Abbreviation	Supplier
Layer A	HP525j	3n	LyondellBasell
	HP501l	6n	LyondellBasell
Layer B	QCPP	16r	LyondellBasell
	QCPT	50r	LyondellBasell

**Table 2:** Thickness ratios, corresponding melt pump setting, extruded sheet configurations.

	A100 B0	A70 B30	A50 B50	A30 B70	A0 B100
Melt pump A in rpm Melt pump B in rpm	54 0	38 16	27 27	16 38	0 54
Shall thickness layer A in µm Shall thickness layer B in µm	550 0	385 165	250 250	165 385	0 550
3n	x				
3n_16r		x	x	-	
3n_50r		x	x	-	
6n	x				
6n_16r		x	x	-	
6n_50r		x	x	-	
16r					x
50r					x

x: extruded sheets. -: extrusion not possible. Materials: 3n (HP525j), 6n (HP501l), 16r (QCP P), 50r (QCP T).

## Data Availability

All data are contained within the article.
